# Experimental data on mechanical properties evaluation of medium carbon steel quenched in different waste media

**DOI:** 10.1016/j.dib.2018.08.185

**Published:** 2018-09-05

**Authors:** P.P. Ikubanni, O.O. Agboola, A.A. Adediran, A.A. Adeleke, B.T. Ogunsemi, T.S. Olabamiji, D.C. Uguru-Okorie, C.O. Osueke

**Affiliations:** a Department of Mechanical Engineering, College of Engineering, Landmark University, PMB 1001, Omu-Aran, Kwara State, Nigeria; b Department of Mechanical Engineering, Faculty of Engineering and Technology, University of Ilorin, Ilorin, Kwara State, Nigeria

**Keywords:** Mechanical, Tensile strength, Yield strength, Quenching media, Hardness value, Properties

## Abstract

Several wastes can be instrumental in the improvement of the mechanical properties of medium carbon steel when quenched. The quenching media employed such as coconut water (CW), pap water (PW) and spent engine oil (SPE) have been largely considered as wastes. The data in this article are related to the research article titled “Mechanical Properties Improvement Evaluation of Medium Carbon Steels Quenched in Different Media” (Ikubanni et al., 2017) [1]. The article provides information on the mechanical properties evaluation of medium carbon steel quenched in different media. Twenty-seven (27) samples of medium carbon steel samples were heated to temperatures of 730 °C, 760 °C and 790 °C and soaked for 30, 45 and 60 min respectively. The test results recorded include hardness value, yield strength (YS) and the ultimate tensile strength (UTS) for each of the samples at different heating temperatures and soaking time for the different quenching media.

## Specifications table

**Table:** 

Subject area	Mechanical Engineering
More specific subject area	Material Engineering, Construction Materials, Waste Management
Type of data	Tables, Figures
How data was acquired	Preparing medium carbon steel samples in the laboratory and applying load to obtained hardness value, yield strength and ultimate tensile strength.
Data format	Raw, processed, and analyzed
Experimental factors	The prepared samples were heated to temperature 840 °C and normalized. During the soaking process, the medium carbon steel samples were left in the furnace for even distribution of temperature. The specimens were tested at laboratory conditions.
Experimental features	Samples prepared were quenched in different media and subjected to hardness and tensile tests in the laboratory.
Data source location	Data obtained from the strength of material laboratory, Landmark University, Omu-Aran.
Data accessibility	All the data are in this article as presented.

## Value of the data

• The data presented shows that the three different quenching media used increased the mechanical properties of the medium carbon steel.
• The data gives information that substances/materials considered as waste can be utilized in improving the mechanical properties of medium carbon steel, and could be considered useful for construction purposes.
• The data provides information on the hardness value, yield strength and ultimate tensile strength of each of the medium carbon steel quenched between the temperature range of 730 °C and 790 °C.

## 1 Data

The data presented information on the hardness value, yield strength and ultimate tensile strength of medium carbon steel samples. The chemical composition of the medium carbon steel under consideration is as shown in Table 1.

**Table 1:** Chemical composition of the medium carbon steel.

**C**	**Si**	**Mn**	**S**	**Cr**	**Ni**	**Cu**	**Fe**
**0.389**	0.182	0.980	0.030	0.111	0.135	0.368	97.805

The hardness value, yield strength and ultimate tensile strength obtained when quenched in CW, PW and SPE are as shown in Table 2, Table 3, Table 4; at different heating temperatures (HT) and different soaking time (ST).

**Table 2:** Mechanical properties of samples quenched in coconut water.

S/N	HT (°C)	ST (min)	Hardness (BHN)	YS (MPa)	UTS (MPa)
0	control	–	166.40	162.9	171.1
1	730	30	349.19	220.0	252.6
2	760	30	332.27	224.1	248.5
3	790	30	332.27	468.5	499.0
4	730	45	269.91	207.8	228.1
5	760	45	609.97	264.8	268.8
6	790	45	573.35	382.9	391.1
7	730	60	499.23	89.6	93.7
8	760	60	573.35	183.3	191.0
9	790	60	573.35	358.5	399.2

**Table 3:** Mechanical properties of samples quenched in pap water.

S/N	HT (°C)	ST (min)	Hardness (BHN)	YS (MPa)	UTS (MPa)
1	730	30	296.91	301.5	334.1
2	760	30	499.23	224.1	248.5
3	790	30	471.59	224.1	252.6
4	730	45	349.19	116.1	138.5
5	760	45	573.35	171.1	183.3
6	790	45	609.97	268.9	317.8
7	730	60	367.30	277.0	285.2
8	760	60	532.37	407.4	427.8
9	790	60	499.23	342.2	350.4

**Table 4:** Mechanical properties of samples quenched in spent engine oil.

S/N	HT (°C)	ST (min)	Hardness (BHN)	YS (MPa)	UTS (MPa)
1	730	30	210.61	529.6	539.8
2	760	30	243.48	236.3	277.0
3	790	30	283.30	382.9	415.5
4	730	45	219.85	158.9	175.2
5	760	45	440.37	309.6	342.2
6	790	45	311.41	317.8	342.2
7	730	60	332.27	342.2	399.2
8	760	60	296.91	382.9	391.1
9	790	60	393.52	301.5	325.9

## 2 Experimental design, materials and methods

**Fig. 1:**
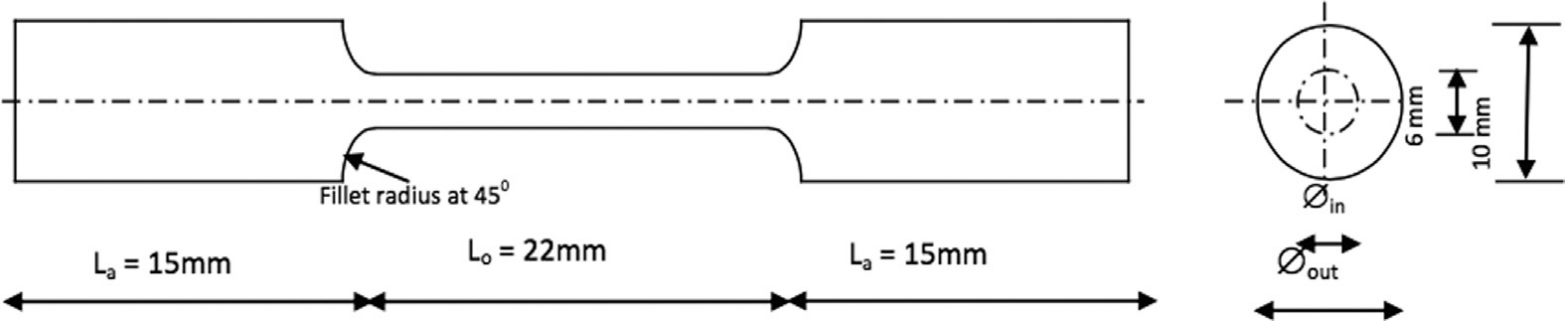
Tensile test specimen.

**Fig. 2:**
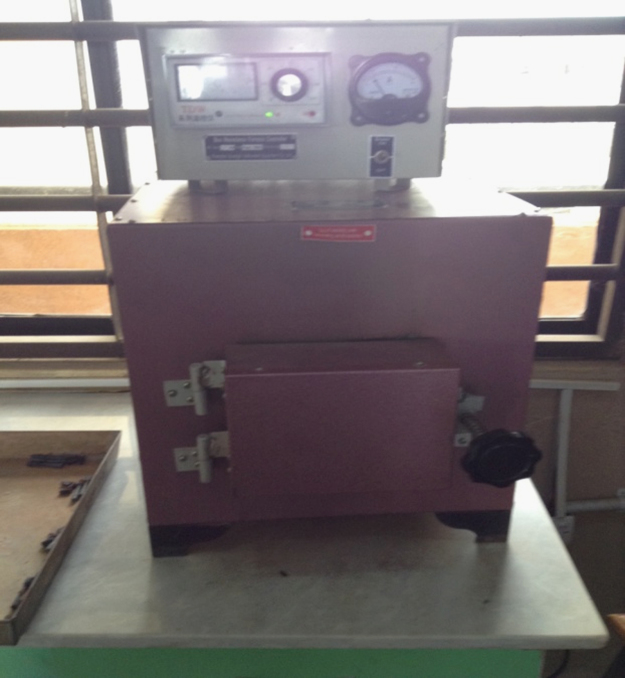
Muffle furnace.

**Fig. 3:**
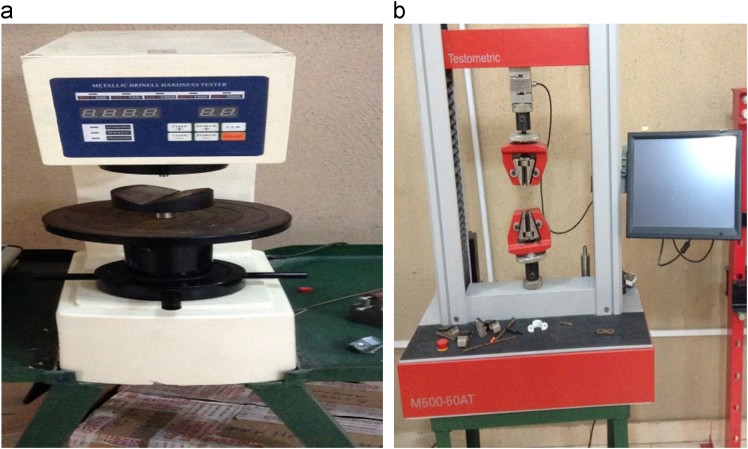
a: Brinell hardness testing machine. b: Testometric machine for tensile test.

The medium carbon steel used for this experiment was obtained from a local market in Omu-Aran, Kwara State (**Latitude:** 8°08׳18.85" N, **Longitude:** 5°06׳09.36" E). The chemical composition of the medium carbon steel was determined using optical emission spectrophotometer. The quenching media (considered as wastes) utilized for the quenching include coconut water (CW), pap water (PW) and spent engine oil (SPE). Medium size lathe machine was used in preparing the samples for tensile test as shown in Fig. 1. In order to reduce the stresses that might have been induced during machining operations on the samples, muffle furnace was used to heat-treat the samples to temperature of 840 °C and then normalized. This was also done for structural and metallurgical re-adjustment, re-conditioning of the phases and induction of homogeneous structure in the samples. A control sample and twenty-seven (27) samples were obtained for the experiments. The control sample was only normalized so as to serve as the basis for comparison. The prepared samples were later heated to 730 °C, 760 °C and 790 °C and soaked for 30, 45 and 60 min, respectively, using a muffle furnace (Fig. 2) in batches. After each heat treatment temperature have been reached, the samples were quickly removed from the furnace and quenched in the different media; which were coconut water, pap water and spent engine oil. The control sample and the quenched samples were subjected to hardness test using Metallic Brinell hardness tester (Model/Serial No.: EEDB 0006/13) (Fig. 3a) for the determination of the hardness value of the different quenched samples together with the control sample. A Testometric M500-50AT model machine (Fig. 3b) was used for the tensile test. The yield and tensile strength were determined for each of the sample used according to Ikubanni et al., 2017 [1].
